# *Lycium barbarum* polysaccharide improves dopamine metabolism and symptoms in an MPTP-induced model of Parkinson’s disease

**DOI:** 10.1186/s12916-022-02621-9

**Published:** 2022-10-28

**Authors:** Jiangbo Song, Lian Liu, Zhiquan Li, Ting Mao, Jianfei Zhang, Lei Zhou, Xin Chen, Yunzhu Shang, Tao Sun, Yuxin Luo, Yu Jiang, Duan Tan, Xiaoling Tong, Fangyin Dai

**Affiliations:** 1 State Key Laboratory of Silkworm Genome Biology, Key Laboratory for Sericulture Biology and Genetic Breeding, Ministry of Agriculture and Rural Affairs, College of Sericulture, Textile and Biomass Sciences, Southwest University, Chongqing, 400715 China; 2 Center for Healthy Aging, Department of Cellular and Molecular Medicine, University of Copenhagen, 2200 Copenhagen N, Denmark

**Keywords:** Parkinson’s disease, Animal model, Silkworm, *Lycium barbarum* polysaccharide, Dopamine, Oxidative stress

## Abstract

**Background:**

Parkinson’s disease (PD) is the second most common neurodegenerative disease in middle-aged and elderly populations, whereas there is no cure for PD so far. Novel animal models and medications await development to elucidate the aetiology of PD and attenuate the symptoms, respectively.

**Methods:**

A neurotoxin, 1-methyl-4-phenyl-1,2,3,6-tetrahydropyridine (MPTP), was used in the current study to establish a PD pathologic model in silkworms. The time required to complete specific behaviours was recorded. Dopamine content was detected by ultra-performance liquid chromatography (UPLC). The activity of insect tyrosine hydroxylase (TH) was determined using a double-antibody sandwich method. Oxidative stress was assessed by changes in antioxidant enzyme activity and the content of oxidative products.

**Results:**

MPTP-treated silkworms were characterized by impaired motor ability, reduced dopamine content, and elevated oxidative stress level. The expression of TH, a dopamine biosynthetic enzyme within dopaminergic neurons in the brain, was significantly reduced, indicating that dopaminergic neurons were damaged. Moreover, MPTP-induced motility impairment and reduced dopamine level in the silkworm PD model could be rescued after feeding a combination of levodopa (L-dopa [LD]) and carbidopa (CD). MPTP-induced oxidative damage was also alleviated, in ways consistent with other PD animal models. Interestingly, administration of *Lycium barbarum* polysaccharide (LBP) improved the motor ability, dopamine level, and TH activity, and the oxidative damage was concomitantly reduced in the silkworm PD model.

**Conclusions:**

This study provides a promising animal model for elucidating the pathogenesis of PD, as well as a relevant preliminary drug screening (e.g., LBP) and evaluation.

## Background

Parkinson’s disease (PD) is the second most common neurodegenerative disease after Alzheimer’s disease. PD is characterized by a large number of dyskinetic and non-dyskinetic features, including motor dysfunctions (such as resting tremor, rigidity, bradykinesia, and postural instability) and non-motor symptoms (such as autonomic dysfunction, cognitive/neurobehavioural abnormalities, sleep disorders, and paresthesias) [1, 2]. Ageing is the most important risk factor for PD, and biochemical changes resulting from ageing amplify the abnormalities in the brain of patients with PD [3]. The incidence of PD increases with age, with > 12 million people expected to be diagnosed with PD by 2040 [4, 5]. The main pathologic features of PD are the degeneration and death of dopaminergic neurons in the substantia nigra and a significant reduction in dopamine content [6, 7]. Although displaying phenotypic similarity, PD is classified as familial (85–90%) and sporadic (10–15%) based on the aetiology and pathogenesis [8, 9]. Familial PD is commonly caused by alterations in six genes: membrane interaction gene (*alpha*-*synuclein* [*SNCA*]); mitochondrial quality control genes (*parkin* [*PRKN*], *PTEN*-*induced kinase 1* [*PINK1*], and *leucine*-*rich repeat kinase 2* [*LRRK2*]); oxidative damage control gene (*Parkinson disease protein 7* [*PARK7*]); and lysosomal enzyme gene (*glucocerebrosidase* [*GBA1*]) [9, 10]. The aetiology and pathogenesis of sporadic PD are more complicated owing to diverse genetic and environmental factors. Both environmental and genetic factors can lead to the degeneration and death of dopaminergic neurons. Mitochondrial dysfunction, oxidative stress, altered protein processing, and inflammatory changes are currently considered to be the primary causes of neuronal dysfunction and death through apoptosis [11].

The treatment of PD patients focuses on the improvement of motor disorders (such as tremor, stiffness, and bradykinesia) and non-motor disorders (such as constipation, cognition, mood, and sleep) [12]. Potential treatment strategies for PD include drug therapy, gene therapy, stem cell transplantation, surgery, and deep brain stimulation [13–15]. Most patients with PD adopt drug therapy; gene- and cell-based treatments have not achieved great breakthroughs in therapy. Medications include L-dopa (LD), dopamine agonists, anticholinergics, amantadine, monoamine oxidase inhibitors, and catecholamine-O-methyltransferase inhibitors [16, 17]. Regular use of LD is beneficial to patients with PD in the long term [18]. Dopamine agonists reduce adverse motor fluctuations, but also have side effects that decrease the quality of life in PD patients and pose a great risk to elderly patients with cardiovascular disease [19]. Anticholinergic drugs can only be used in patients with early-stage PD, inhibit central acetylcholine activity, but induce memory and cognitive dysfunction [20]. Amantadine was initially used for the treatment of influenza and has gradually been used in patients with early-stage PD, but amantadine is poorly tolerated in elderly PD patients [21]. Monoamine oxidase inhibitors have a good pharmacokinetic profile that improves the state of dopamine deficiency and results in a significant improvement in dyskinesia [22]. Moreover, peripheral catecholamine-O-methyltransferase inhibitors reduce the systemic metabolism of LD, but some drugs need to be combined with LD to have an effect [23]. As the disease progresses, there is an emergence of complications related to long-term symptomatic treatment, including motor and non-motor fluctuations, dyskinesia, and psychosis [24–28].

Widely used PD animal models contribute to the accumulation of evidence regarding the molecular mechanism and pathogenesis of PD. At present, the modelling of PD mainly includes transgenic and neurotoxin-induced models. Mutations in the *PRKN* gene, which encodes an E3 ubiquitin-protein ligase, are the most common cause of recessive PD and early-onset PD by blocking protein degradation and causing protein aggregation, resulting in neurotoxicity [29]. This finding serves as the basis for *PRKN*-targeted knockout familial PD models. A variety of neurotoxins can mimic PD models, such as 6-hydroxydopamine (6-OHDA), rotenone, reserpine, maneb, and paraquats [30–33]. The 6-OHDA, a catecholaminergic neurotoxin, cannot cross the blood-brain barrier; therefore, it needs to be injected into the substantia nigra of the brain. Moreover, this method is unable to completely recapitulate the symptoms of PD patients, and it does not form the features of PD (Lewy bodies) [34, 35]. Rotenone is a fat-soluble herbicide that passes through the blood-brain barrier, but selectively inhibits mitochondrial respiratory chain complex I, causing multiple organ damage and high mortality in animals [36]. Reserpine is an inhibitor of the vesicular monoamine transporter 2 in the central nervous system that reduces the content of monoamines and is used as an effective antihypertensive drug. Large doses of reserpine also cause PD-like symptoms in experimental animals, but it does not cause persistent neuronal damage [37]. The chemical structure of paraquat is similar to 1-methyl-4-phenylpyridinium (MPP^+^), a commonly-used herbicide, and maneb, which is a fungicide. The combined use of these two drugs can better simulate the pathogenesis of PD patients; however, there are few studies involving the adverse effects of paraquat and maneb, and as a result, it is not easy to evaluate the safety of the combined use of paraquat and maneb [38]. The 1-methyl-4-phenyl-1,2,3,6-tetrahydropyridine (MPTP)-induced PD models reproduce the pathologic features of PD at multiple levels, and no severe supererogatory side effects have been found, so MPTP is ideal for PD modelling [39, 40].

Oxidative damage to endogenous macromolecules is known to lead to the excess accumulation of free radicals, which triggers a cascade of events leading to cell death [41]. PD patients often exhibit oxidative damage. Previous research has shown that flavonoids, saponins, polysaccharides, alkaloids, and other substances in nature have antioxidant and anti-ageing effects [42, 43]. These substances provide a theoretical scope for targeted screening of potential therapeutic drugs for PD. *Lycium barbarum* polysaccharide (LBP) is a proteoglycan that has anti-ageing and anti-oxidation, reducing blood lipid and enhancing immunity functions. LBP has been reported to have neuroprotective effects against cerebral reperfusion-induced injury in the brain by scavenging free radicals [44]. LBP upregulates levels of superoxide dismutase 2 (SOD2), catalase (CAT), and glutathione peroxidase 1 (Gpx1) and inhibits the abnormal aggregation of α-synuclein induced by MPTP [45]. In addition, LBP plays an important role in protecting against neurotoxicity via the *Nrf2*-*HO*-*1* pathway [46]. Furthermore, LBP protects optic neurons after treatment in a mouse glaucoma model [47]. Therefore, we hypothesize that LBP might protect dopaminergic neurons from the pathologic symptoms of PD.

In this study, a novel PD animal model was established using MPTP in silkworms. This model was then utilized to investigate dopamine metabolism and oxidative stress. Restoration was achieved by LD and CD combination and enhanced by the drug efficacy of a small molecule called LBP.

## Methods

### Silkworm feeding and body weight determination

The silkworm wild-type strain was obtained from the Silkworm Gene Bank at Southwest University (Chongqing, China). The silkworm larvae were maintained at 25.5 ± 1 °C with a relative humidity of 90–65% with circadian rhythms of 12 h light/12 h dark each day of the life cycle. The silkworms were reared ad libitum with fresh mulberry leaves throughout the larval stage [48]. The larval body weights of silkworms were measured from the 2nd instar before feeding the mulberry leaves each day. Three sets of biological replicates were designed for each group with 10 silkworm individuals. This study conformed to the statement of Southwest University on the Welfare of Animals.

### Toxicity of MPTP to silkworm individuals

This experiment adopted the method that mulberry leaves were smeared with medicaments for feeding. Three concentrations of MPTP solution (125, 250, and 500 μM) were spread on the back of mulberry leaves, and phosphate-buffered saline (PBS) solution was used as a control. Each group contained 100 silkworm larvae, fed with the corresponding smeared mulberry leaves that had dried. The daily intake of medicaments in the four groups was as follows: 1 μL for 2nd instar silkworms, 2 μL for 3rd instar silkworms, 5 μL for 4th instar silkworms, and 20 μL for 5th instar silkworms. The silkworms were fed on mulberry leaves smeared with MPTP or PBS solution in the morning, and common mulberry leaves for the remainder of the time. Finally, the toxic effect of different concentrations on silkworms was determined according to the number of surviving silkworms on day 5 of the 5th instar larvae.

### Determination of silkworm behaviour

The movement speed was measured by the observation and analysis system of insect behaviour and habits (Beijing CaSO Zhonghe Technology Co., Ltd., Beijing, China). The temperature and humidity conditions were set at 25 °C and 75%, respectively. Five 5th instar larvae were placed into the instrument and the behaviour trajectory was recorded within 5 min. The movement speed was subsequently calculated according to the movement area within 5 min. The turnover ability was determined by recording the period required for the silkworm from turning over to returning to the normal posture. The grasping ability of the silkworm was detected by recording the period required for the silkworm from being put on the brush holder to falling. The abdominal bending ability was detected by turning the silkworm over with the head pressed, then recording the period until the silkworm’s abdomen bent 90°.

### Determination of total antioxidant activity and oxidation product content

Silkworm individuals were dissected and the brain tissues were obtained. The brain tissues were washed twice with PBS solution and immediately quick-frozen with liquid nitrogen. Tissues were ground into a smooth fine powder, then packed into 1.5-mL RNase-free centrifuge tubes and quick-frozen to prepare protein samples. A BCA protein concentration determination kit (Beyotime Biotech Inc, Shanghai, China) was used to determine the protein concentration. The activity of total antioxidant capacity and malondialdehyde (MDA) level was assayed using kits (Suzhou Keming Biotechnology Co., Ltd., Suzhou, China). Additionally, the homogenate content from an entire silkworm brain was assayed according to the kit instructions.

### Detection of tyrosine hydroxylase (TH) activity

The activity of insect TH was determined using a double-antibody sandwich method with an insect TH enzyme-linked immunosorbent assay kit (Jiangsu Enzyme Immune Industrial Co., Ltd., Yancheng, China). The sample was ground into powder and fully dissolved with PBS to determine the protein concentration. Fifty-microliter standards were added in the control group, while 40-μL sample diluent and 10-μL sample were added in the treatment group, mixed well, and the plate was sealed. Standards and sample mixtures were incubated for 30 min at 37 °C and washed five times. Then, 50-μL enzyme-linked immunosorbent assay reagent was added. Standards and sample mixtures were incubated for 30 min at 37 °C after washing five times. The following colour development step was performed according to the kit instructions. The absorbance was measured at 450 nm within 15 min after termination of the reaction. The protein activity of the sample was calculated according to the standard curve.

### Detection of dopamine content by ultra-performance liquid chromatography (UPLC)

The brain tissues dissected from day 5 of the 5th instar silkworms fed with 125 μM MPTP and PBS were preserved in dopamine extract and stored in the dark. Five samples of brain tissue were taken from each of the four groups. Tissue grinding was performed on ice with a hand-held homogenizer (MY-10, Shanghai Jingxin Industrial Development Co., Ltd., Shanghai, China).

The ground homogenate was centrifuged for 10 min at 4 °C and 12,000 rpm to extract dopamine from the sample. The supernatant was transferred to a new 1.5-mL RNase-free centrifuge tube (wrapped with tinfoil paper) and boiled for 5 min to denature proteins. After the supernatant was cooled to room temperature, two volumes of methylene chloride were added as the deproteinizing solution, vortexed for 3 min, and centrifuged for 10 min at 4 °C and 12,000 rpm. The supernatant was obtained for UPLC (LC-30A, Shimadzu, Kyoto, Japan) analysis.

The content of dopamine was determined by UPLC with Shim-pack XR-ODS III (2.0 mm × 75 mm, 1.6μm, Shimadzu, Kyoto, Japan). Labsolution software (Shimadzu, Kyoto, Japan) was used and the RF-20a detector was selected. The automatic exhaust mode was set for flow paths A, B, C, and D for 5 and 10 min for R0 and R3, respectively. The mobile phase consisted of 0.1 M citrate phosphate buffer (pН 2.68), containing 0.3 mM sodium octanesulfonate (Sigma, St Louis, USA), 0.1 mM EDTA (Sangon Biotech (Shanghai) Co., Ltd., Shanghai, China), and 10% methanol (Sigma, St Louis, USA). The parameters were set as follows: data acquisition time, 5 min; injection volume, 5 μL; injection needle stroke of the autosampler, 50 mm; sample aspiration rate, 5 μL/sec; flow rate, 0.2 mL/min; and chromatographic column oven temperature, 40 °C. The content of dopamine in the sample was calculated according to the standard curve.

### Preparation of brain tissue section

The brain tissues of the silkworms were dissected under a microscope. The brain tissues were fixed with 4% paraformaldehyde for 10–20 min and washed once with PBS. The fixed brain tissues were embedded in the OCT embedding agent (SAKURA, Japan) and placed in a freezer for 30–60 min at −80 °C. The embedded tissue samples were placed in a frozen microtome for sectioning. The sectioned brain tissues were taken with an anti-falling glass slide and placed at room temperature for 30 min. The brain tissues were sealed and stored at −20 °C, but not treated immediately.

### Immunofluorescence

All sections were incubated under standard conditions as follows: (a) in 4% paraformaldehyde (Beyotime Biotech Inc, Shanghai, China) for 5–10 min at room temperature; (b) in sodium citrate antigen retrieval solution (Beyotime Biotech Inc, Shanghai, China) treated under microwave at low power heat for 10 min; (c) in seal solution (Beyotime Biotech Inc, Shanghai, China) for 1 h at room temperature or 24 h at 4 °C; (d) TH-polyclonal antibody (1:500; Shanghai Youke Biotechnology Co. Ltd., Shanghai, China) for 24 h at 4 °C; and (e) CY3-labelled goat anti-rabbit IgG H + L (1:100; Beyotime Biotech Inc, Shanghai, China) for 1–2 h at room temperature. The sections were washed three times with PBS after each incubation step for 5 min each. After the sections were dehydrated, the DAPI-containing anti-fluorescence quencher was added dropwise. The sections were incubated with a cover glass in the dark for 10 min. Nail polish was dropped at the edge of the coverslip to prevent slippage, and the sections were imaged with a fluorescence microscope (BX63; Olympus Corporation, Tokyo, Japan).

### Real-time quantitative PCR (RT-qPCR)

The CDS sequences of corresponding genes were introduced into Primer 5.0 software (Premier, San Francisco, USA). The primers were designed to make the length of the amplified products between 80 and 350 bp. Total RNA was extracted using a Total RNA Kit (BioTeke Corporation, Beijing, China), and the cDNA was synthesized using a PrimeScript^TM^ RT Reagent Kit with gDNA Eraser (Takara Biomedical Technology (Beijing) Co., Ltd., Beijing, China). RT-qPCR was performed on qTOWER^3^ G (analytikjena, Jena, Germany). The procedure was set according to the protocols of the SYBR Green Supermix Kit (Yeasen Biotechnology (Shanghai) Co., Ltd., Shanghai, China). Primer sequences and their targeted genes are shown in Table 1. The relative expression of each gene was normalized to the reference gene, *sw22934*, and calculated as 2^−△△CT^ [49].

**Table 1:** The primer sequences for RT-qPCR used in this study

Gene name	Forward primer (5′-3′)	Reverse primer (5′-3′)
*sw22934*	TTCGTACTGCTCTTCTCG	CAAAGTTGATAGCAATTCCCT
*BmTH*	TTCAGGACTGAACACAAACTCT	GGTTCTGCGGTTCTTTATCATC
*BmPARK2*	GAAGATGACACGAAAGACGATG	CTCAAGCTCAGTTTGTCTTTCC
*BmDDC*	TGCGGTGATGGCGGATAT	TGGGAAGTAGGCGTGGAAC

### Statistical analysis

Statistical analyses were performed using GraphPad Prism 9.0 (GraphPad Software, San Diego, USA). The differences in independent experiments were analysed and calculated using a two-tailed Student’s *t*-test and two-way ANOVA. The error bars are presented as the mean ± SD and *p* < 0.05 was considered statistically significant.

## Results

### PD silkworm model establishment and phenocopy

**Fig. 1:**
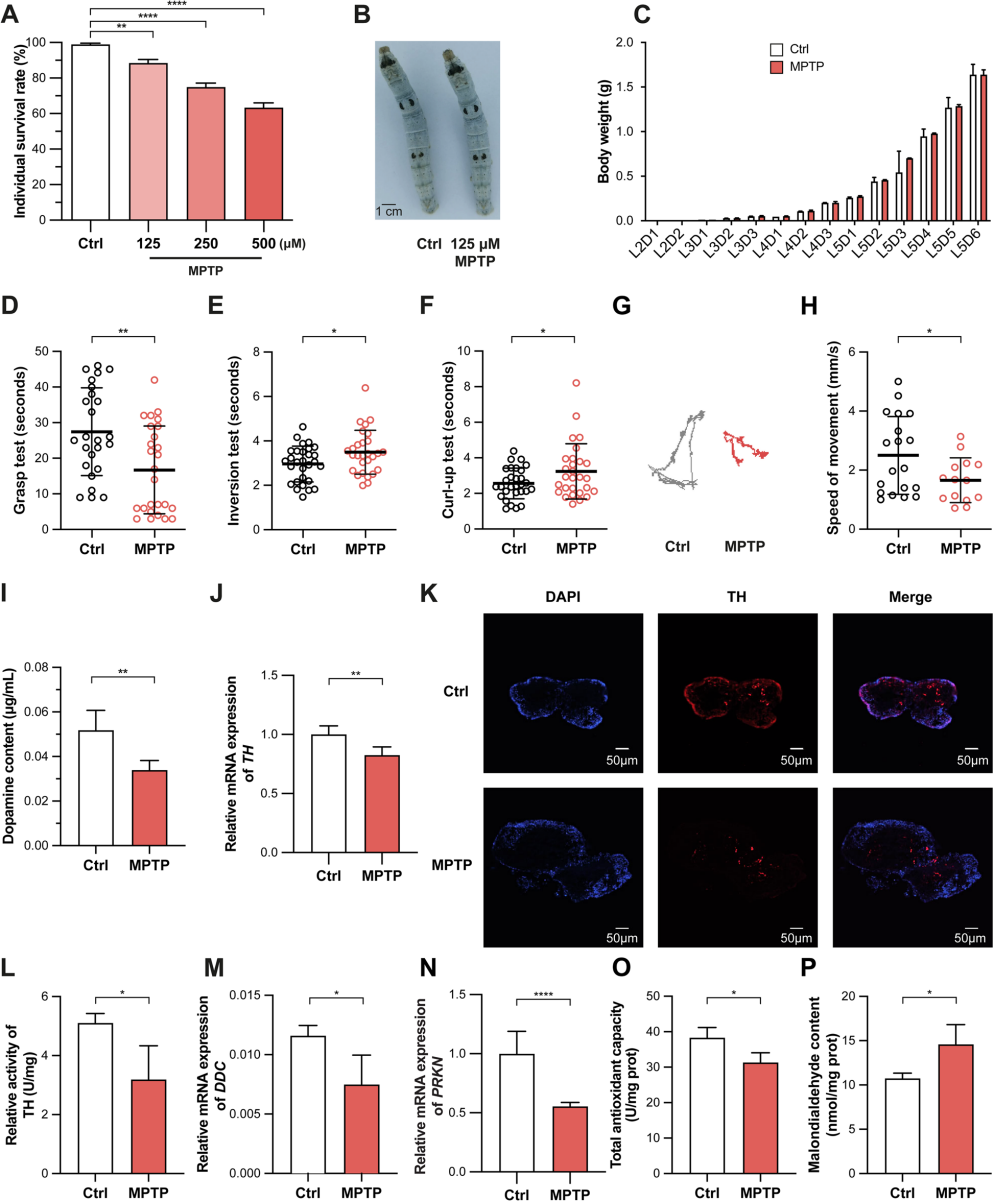
Establishment and evaluation of the PD model in silkworms. **A** Detection of toxicity among different concentrations of MPTP in silkworms by CCK8 (125, 250, and 500 μM, *n* = 100/concentration). **B** A photographic comparison of the morphology of untreated silkworms and MPTP-treated (125 μM) silkworms. **C** The average body weight in different larval stages (*n* = 30/stage). **D–H** A comparison of locomotor capacity between wild-type and PD silkworms (*n* = 30/condition). **I** Detection of dopamine content between untreated and MPTP-treated (125 μM) silkworms by UPLC (*n* = 4). **J** Detection of *BmTH* gene expression in silkworm brain tissues by qPCR (*n* = 4). **K** Detection of TH protein expression in silkworm brains by immunofluorescence. **L** Detection of BmTH activity in silkworm brains by enzyme-linked immunosorbent assay. **M**, **N** Detection of relative expression of *BmDDC* and *BmPARK2* in silkworm brains by RT-qPCR (*n* = 4). **O**, **P** Determination of antioxidant capacity and oxidation product content in brains of day 5 of the 5th instar larvae (*n* = 4). All the experiments were performed at least 3 times and showed a similar pattern. Data are presented as the mean with SD and analysed using Student’s *t*-test: *p < 0.05, **p < 0.01, ****p < 0.0001

To model PD in silkworms, wild-type silkworms were fed with different concentrations of a neurotoxin (MPTP). MPTP administration showed a negative correlation between the dose and individual survival rate, with the highest survival at 125 μM compared to 250 and 500 μM (Fig. 1A). The body size and morphology of silkworms did not change significantly after feeding 125 μM MPTP (Fig. 1B). Moreover, the average body weight of silkworms fed with 125 μM MPTP was not significantly different from the control group (Fig. 1C). Therefore, due to the low toxicity and normal development, 125 μM MPTP was used to model PD thereafter. To further assess the capability of MPTP in inducing PD-like symptoms, the motor ability was measured by on-spot movement (grasp, inversion, and curl-up) and free movement. On-spot movement in PD silkworms showed a shorter grasping time, slower inversion/turning speed, and more moderate curl-up/abdominal bending (Fig. 1D–F). The locomotor activity of silkworms was determined by the insect behaviour trajectory recorder and showed a significantly shorter movement path and slower crawling speed after feeding with MPTP (Fig. 1G, H).

A significant decrease in the neurotransmitter dopamine content in the brain is an important clinical diagnostic marker in PD patients. To investigate the dopamine metabolism in the MPTP-induced PD model, we first checked the dopamine content in the brain and found a dramatic decrease in PD silkworms (Fig. 1I). Then, we asked whether this resulted from an inhibition of the upstream TH, a key catalytic enzyme in the dopamine synthesis pathway. We found that the level of the *TH* gene expression was significantly downregulated (Fig. 1J). Concomitantly, it was found that the level of TH protein expression within dopaminergic neurons in the brain of silkworms fed with MPTP was significantly reduced, as detected by brain tissue section immunofluorescence (Fig. 1K). Strikingly, the BmTH activity of the silkworm brain was also significantly reduced (Fig. 1L). To further investigate the genes downstream of TH in dopamine metabolism, the level of *dopa decarboxylase* (*DDC*) expression, which catalyses the synthesis of dopamine from LD, was also measured, and the results showed its expression was significantly reduced (Fig. 1M), consistent with a decrease in dopamine content. PRKN, an E3 ubiquitin-protein ligase, protects dopaminergic neurons and inhibits oxidative stress, and its mutations cause juvenile PD [50]. Therefore, the level of mRNA expression was determined, and we found a marked decrease in *PRKN* within the silkworm brain after feeding with MPTP (Fig. 1N).

Moreover, elevated oxidative stress is widely observed in PD beyond a reduced dopamine level [51]. Then, the oxidative status was detected in the brains of silkworms fed with MPTP. The results showed that the total antioxidant capacity of the fed silkworms was significantly reduced, and the content of MDA, an oxidative stress marker, was significantly increased (Fig. 1O, P). Together, these results proved that the phenotype of silkworms treated with MPTP was highly similar to many clinical symptoms of PD in humans, indicating that the silkworm model induced by MPTP served as a promising PD animal model.

### Evaluation of the therapeutic effect of LD therapy on the PD silkworm model

**Fig. 2:**
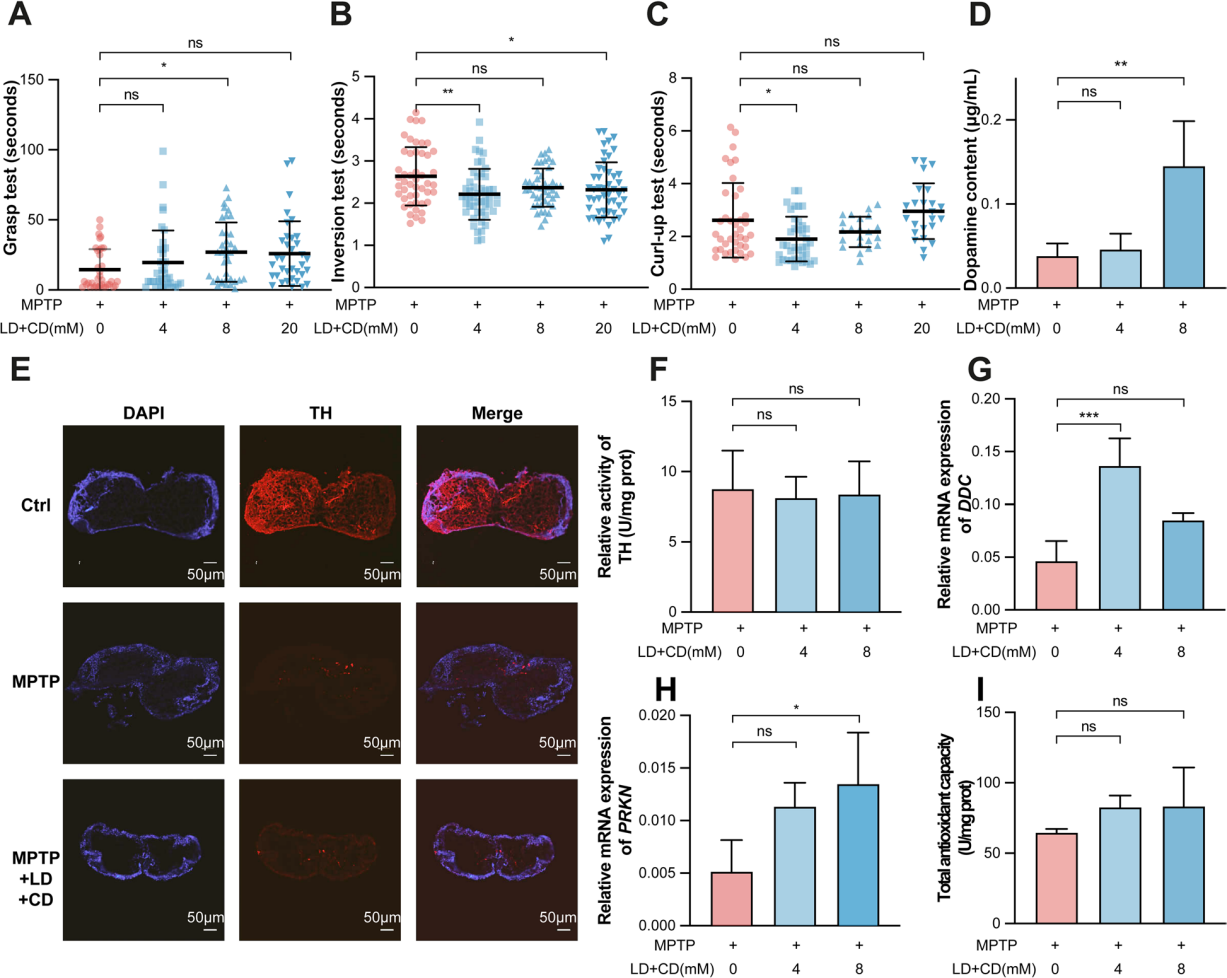
Evaluation of the therapeutic effect of LD-CD mixture on the PD model. **A–C** Detection of the locomotor capacity of day 5 of the 5th instar larvae treated by MPTP (125 μM) with LD-CD mixture (0, 4, 8, and 20 mM; *n* = 5/locomotor situation). **D** Determination of dopamine content in PD silkworm brains with LD-CD mixture by UPLC (0, 4, and 8 mM; *n* = 4/concentration). **E** Detection of TH protein expressions by immunofluorescence among untreated silkworm brains, MPTP-treated (125 μM) silkworm brains, and brains of silkworms treated with MPTP (125 μM) and the LD-CD mixture (8 mM). **F** Determination of BmTH activity by enzyme-linked immunosorbent assay in PD silkworm brains with LD-CD mixture (0, 4, and 8 mM). **G**, **H** Detection of relative levels of *BmDDC* and *BmPARK2* expression by RT-qPCR in brains of silkworms fed with the LD-CD mixture (0, 4, and 8 mM; *n* = 3/concentration). **I** Detection of total antioxidant capacity in silkworms fed with the LD-CD mixture (0, 4, and 8 mM; *n* = 3/concentration). All the experiments were performed at least 3 times and showed a similar pattern. Error bars depicted the mean with SD and were analysed using the Student’s *t*-test. The “ns” is an abbreviation for “no significance”: *p < 0.05, **p < 0.01, ***p < 0.001

To further confirm the feasibility of this newly established PD silkworm model, we administered the clinical drug developed for PD patients (the combination of LD with CD solution) to evaluate the responses of the animal model. When the MPTP-induced PD model was supplemented with a LD-CD mixture solution, the PD silkworms had restored motor activity, significantly improved grasping ability, and faster response in the inversion and curl-up test (Fig. 2A–C). At the same time, dopamine content determination showed that LD-CD significantly restored the dopamine content in the brain of PD silkworms (Fig. 2D). In addition, an immunofluorescence assay using brain tissue sections showed that the reduction in TH protein levels was significantly alleviated after the addition of LD-CD solution, while TH activity was not affected (Fig. 2E, F). To further investigate the recovery of dopamine content, we investigated *DDC* and found that the level of *DDC* expression was significantly increased in the PD model supplemented with LD-CD solution (Fig. 2G). Similarly, the level of *PRKN* expression was also significantly increased (Fig. 2H), while the antioxidant capacity was not affected (Fig. 2I). These results indicated that the LD-CD solution had a significant therapeutic effect on the MPTP-induced PD silkworm model.

### Detection of potential novel therapeutic drugs using the PD silkworm model

**Fig. 3:**
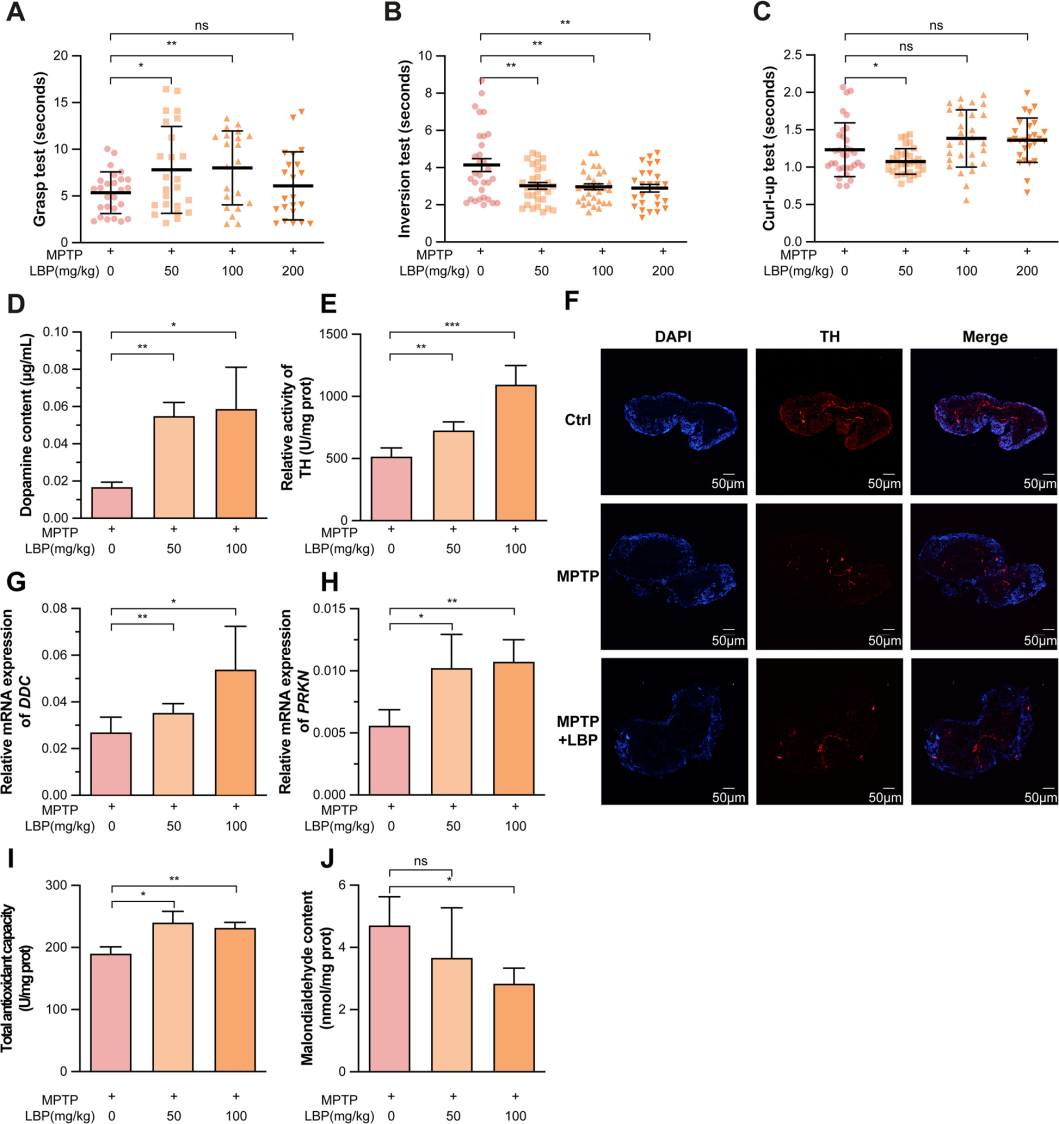
Evaluation of the therapeutic effect of LBP on the PD model. **A–C** Detection of the locomotor capacity of day 5 of the 5th instar larvae fed with LD-CD mixture (4 mM) or LBP (50, 100, and 200 mg/kg; *n* = 5/locomotor situation). **D** Determination of dopamine content in PD silkworm brains with LBP by UPLC (0, 50, and 100 mg/kg; *n* = 4/concentration). **E** Determination of BmTH activity in PD silkworm brains with LBP (0, 50, and 100 mg/kg) by enzyme-linked immunosorbent assay. **F** Detection of TH protein expression among untreated silkworm brains, PD silkworm brains, and brains of PD silkworms with LBP (50 mg/kg) by immunofluorescence. **G**, **H** Detection of the relative levels of *BmDDC* and *BmPARK2* expression in brains of silkworms fed with LBP (0, 50, and 100mg/kg) by qPCR (*n* = 3/concentration). **I**, **J** Detection of total antioxidant capacity and MDA content in PD silkworms fed with LBP (0, 50, and 100 mg/kg; *n* = 3/concentration). All the experiments were performed at least three times and showed a similar pattern. Error bars depicted the mean with SD and were analysed using Student’s *t*-test. The “ns” is the abbreviation for “no significance”: *p < 0.05, **p < 0.01, ***p < 0.001

LBP, a proteoglycan, has been shown to have antioxidant and neuroprotective effects [52]. Specifically, LBP protects PC12 cells against rotenone and 6-OHDA-induced apoptosis [53, 54]. Therefore, we hypothesized that LBP might have similar biological effects on alleviating pathologic features in dopaminergic neurons in PD silkworms. We found that LBP enhanced the grasping, as well as shortened the time required for PD silkworms to turn over and curl-up in 5th instar larvae (Fig. 3A–C). Interestingly, the content of dopamine was significantly increased following LBP administered to PD silkworm, as determined by UPLC in the brains of silkworms (Fig. 3D). The ELISA results showed that TH protein activity of PD silkworms was significantly increased after feeding with LBP (Fig. 3E). This finding was further confirmed by immunofluorescence quantification of the TH protein level showing a restored protein expression from MPTP-mediated downregulation in the brain of PD silkworms (Fig. 3F). Further detection showed that LBP restored the level of mRNA expression of downstream *DDC* in PD silkworms (Fig. 3G). In addition, LBP restored the level of *PRKN* expression in PD silkworms (Fig. 3H). Moreover, the total antioxidant capacity was increased, followed by the decreased content of the oxidative product, MDA, in the brains of the silkworms (Fig. 3I, J). These results indicated that LBP significantly improved the motility and molecular patterns of MPTP-induced PD silkworms similar to the LD and CD combination.

## Discussion

Silkworms contain 5006 genes that are highly homologous to human pathogenic genes [55] and have been developed as models for a variety of human diseases, including sepiapterin reductase deficiency [56], diabetes [57–59], gout [60], epilepsy [61], and other neurodegenerative diseases. As a model of neurodegenerative disease, silkworms have many anatomic and physiologic advantages [62]. First, silkworms have a relatively simple nervous system. Second, silkworms respond sensitively to the stimulation of exogenous substances by both injection and oral administration. Third, there are many mutants of key enzymes in dopamine metabolism in silkworms, such as *TH* mutation (Bmsch mutant) and N-acetyl transferases mutation (Bmmln mutant), among others, which provides convenience for further exploration of dopamine synthesis and metabolic pathways [63].

A recent study used 6-OHDA to induce the PD model in silkworms. Proteomics and metabolomics showed PD-like symptoms, such as locomotion dysfunction, dopamine neuron loss, and alterations in neurotransmitters and structural proteins [64]. Our study chose MPTP to construct a PD model because MPTP is s a typical PD modelling agent due to its penetration properties in the blood-brain barrier. PD animal models of this kind could phenocopy the sporadic PD characteristics, including degeneration of dopaminergic neurons and corresponding motor dysfunction [65]. Oxidative stress is thought to contribute to the pathogenesis of PD by challenging mitochondrial homeostasis, inflammation, and dopaminergic cell death [66]. Therefore, this study also demonstrated systematic oxidative stress along with the dopamine metabolism pathway.

The immediate precursor of dopamine, LD, can be transferred into the brain via a mechanism of facilitated amino acid transport [67]. Orally administered LD is the most common and effective therapy for treating PD due to its curative effect and cost-effectiveness. Except for the dopamine deficiency in PD, the primary therapeutic needs focus on the protection of dopaminergic neurons and the reversal of impaired motor functions. Compared to LD, we found that LBP has similar therapeutic effects in the recovery of locomotor capacity and protection of dopaminergic neurons. Furthermore, LBP has apparent advantages in restoring TH-activity and protecting against the accumulation of oxidative damage in neurodegenerative diseases. Long-term use of a single drug may lead to dependence and side-effects, and a decline in pharmacology response sensitivity. To minimize the risk, using commutative or combined drugs will offer a very efficient and convenient choice. Altogether, LBP might be further investigated by thoroughly measuring the pharmacologic parameters and restored effect in neuroprotection. These results suggest that LBP has been shown to have a potent antioxidative role in alleviating degeneration in dopaminergic neurons, as well as functions in other neurologic disorders.

## Conclusions

**Fig. 4:**
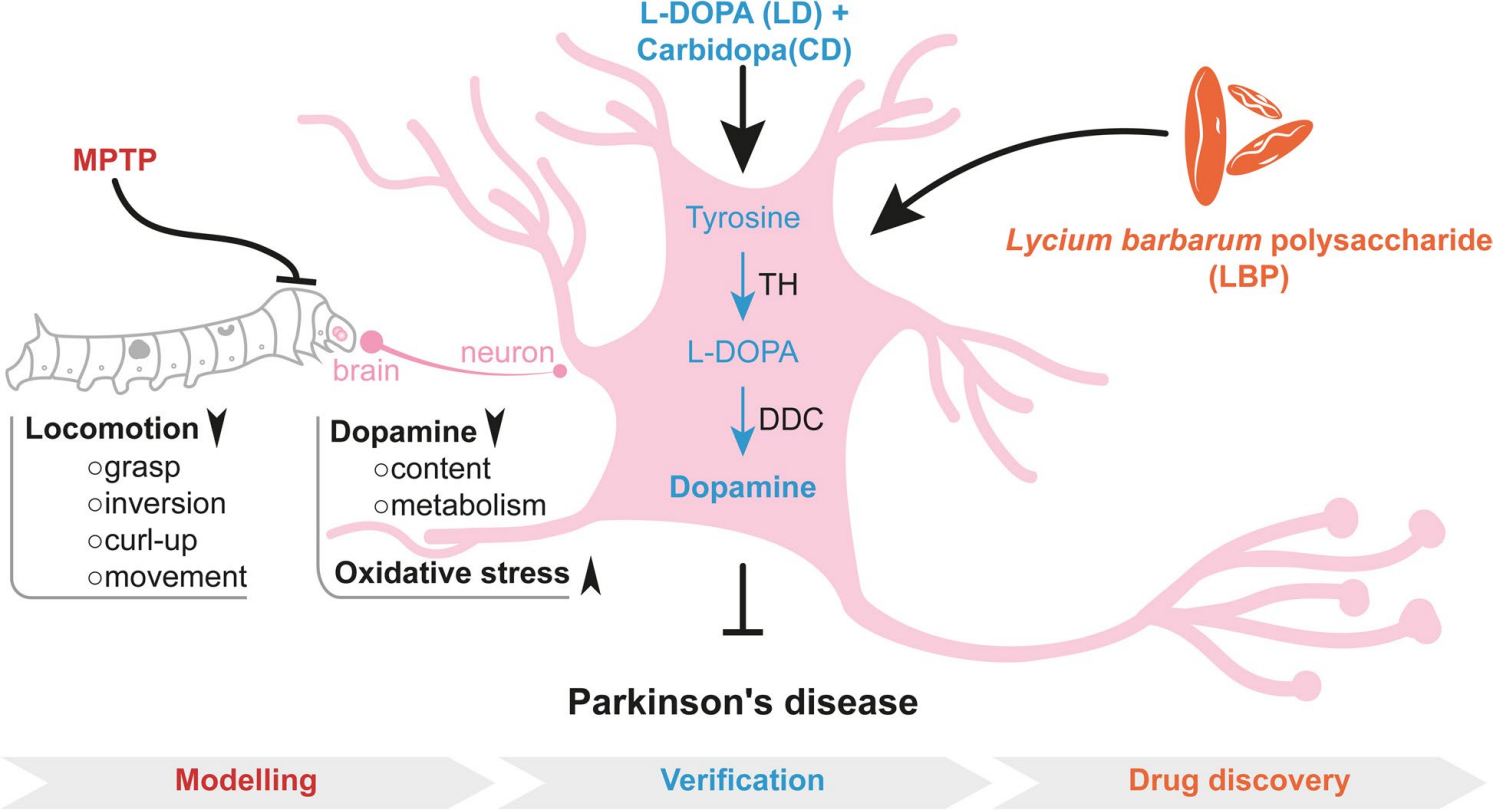
A model illustrating the silkworm PD model construction, evaluation, and application for drug discovery, like LBP

Taken together, we established a novel PD model in silkworms by administering the neurotoxin MPTP. This model showed impaired motor function, decreased dopamine metabolism, and elevated oxidative stress. Supplementation of clinical drugs (LD in combination with CD) alleviated PD symptoms. Interestingly, similar protective effects were also achieved by applying a novel natural molecule, LBP, which enabled normalization of PD-like symptoms and molecular alterations, especially dopamine metabolism and redox homeostasis (Fig. 4). In the future, this model can be used for further studying the aetiology of PD, especially regarding the dopamine metabolism and for the primary drug screening targeting PD in a high-throughput and cost-effective setting.

## Data Availability

All data generated or analysed during this study are included in this published article. Materials generated in this study are available from the corresponding author upon request.

## Abbreviations

Bm: *Bombyx mori*
CAT: Catalase
CD: Carbidopa
DDC: Dopa decarboxylase
*GBA1*: *Glucocerebrosidase 1*
Gpx1: Glutathione peroxidase 1
LBP: *Lycium barbarum* polysaccharide
LD: L-Dopa, levodopa
*LRRK1*: *Leucine*-*rich repeat kinase 2*
MDA: Malondialdehyde
MPP^+^: 1-Methyl-4-phenylpyridinium
MPTP: 1-Methyl-4-phenyl-1,2,3,6-tetrahydropyridine
6-OHDA: 6-Hydroxydopamine
*PARK7*: *Parkinson disease protein 7*
PD: Parkinson’s disease
*PINK1*: *PTEN*-*induced kinase 1*
*PRKN*: *Parkin*
RT-qPCR: Real-time quantitative PCR
*SNCA*: *Alpha*-*synuclein*
SOD2: Superoxide dismutase 2
TH: Tyrosine hydroxylase
UPLC: Ultra-performance liquid chromatography
